# Relational coordination amongst health professionals involved in insulin initiation for people with type 2 diabetes in general practice: an exploratory survey

**DOI:** 10.1186/s12913-014-0515-3

**Published:** 2014-11-01

**Authors:** Jo-Anne Manski-Nankervis, Irene Blackberry, Doris Young, David O’Neal, Elizabeth Patterson, John Furler

**Affiliations:** General Practice and Primary Health Care Academic Centre, University of Melbourne, Carlton, Victoria Australia; Faculty of Health Sciences, La Trobe University, Wodonga, Victoria Australia; Department of Medicine, St Vincent’s Hospital, Fitzroy, Victoria Australia; Department of Nursing, Melbourne School of Health Sciences, University of Melbourne, Carlton, Victoria Australia

**Keywords:** Relational coordination, Insulin initiation, Type 2 diabetes, General practice

## Abstract

**Background:**

The majority of people with type 2 diabetes (T2D) receive their care in general practice and will eventually require initiation of insulin as part of their management. However, this is often delayed and frequently involves referral to specialists. If insulin initiation is to become more frequent and routine within general practice, coordination of care with specialist services may be required. Relational coordination (RC) provides a framework to explore this. The aim of this study was to explore RC between specialist physicians, specialist diabetes nurses (DNEs), generalist physicians in primary care (GPs) and generalist nurses (practice nurses (PNs)) and to explore the association between RC and the initiation of insulin in general practice, and the belief that it is appropriate for this task to be carried out in general practice*.*

**Methods:**

A survey was distributed to a convenience sample of specialist physicians, DNEs, GPs and practice nurses. We collected data on demographics, models of care and RC in relation to insulin initiation. We expected that RC would be higher between specialists than between specialists and generalists. We expected higher RC between specialists and generalists to be associated with insulin initiation in general practice and with the belief that it is appropriate for insulin initiation to be carried out in general practice. We used descriptive statistics and non-parametric tests to explore these hypotheses.

**Results:**

179 health professionals returned completed surveys. Specialists reported higher RC with each other and lower RC with PNs. All groups except PNs reported their highest RC with DNEs, suggesting the potential for DNEs to serve as boundary spanners. Lower RC with specialists was reported by those working within a general practice model of care. Health professionals who felt that a general practice model was appropriate reported lower communication with specialist physicians and higher shared knowledge with GPs.

**Conclusion:**

Given the need for coordination between specialist and generalist care for the task of insulin initiation, this study’s results suggest the need to build relationships and communication between specialist and generalist health professional groups and the potential for DNE’s to play a boundary spanner role in this process.

**Electronic supplementary material:**

The online version of this article (doi:10.1186/s12913-014-0515-3) contains supplementary material, which is available to authorized users.

## Background

Interprofessional care is important for the provision of quality care as it has the potential to provide additional benefits to patients, reduce errors, improve responsiveness, reduce costs and improve the standard of care provided [1-4]. Type 2 diabetes (T2D) is a complex chronic condition where the coordinated efforts of a number of health professionals may be needed to support patients as they manage this lifelong illness.

Type 2 diabetes (T2D) is a health priority because it is common and costly and because of its impact on the burden of illness in the community [5]. T2D is characterized by increased blood glucose levels which result from reduced or less effective insulin. 10 years after diagnosis approximately 50% of people with T2D (PwT2D) will require insulin to maintain glycaemic control [6] and reduce the risk of developing complications, such as damage to the kidneys, eyes and nerves.

### Type 2 diabetes and insulin initiation in general practice

In Australia the majority of clinical care for PwT2D occurs in the general practice setting [7,8]. However, when insulin initiation is eventually needed it is often delayed [9-11] in part because the majority of PwT2D are referred to specialist physicians and diabetes nurse educators (DNEs) [12]. Given the growing prevalence of T2D [13] and the limited availability of these diabetes specialist resources [14,15] making insulin initiation and titration part of routine primary care practice is necessary for uncomplicated diabetes. New models of care to support this are being explored [16-18].

### Health professionals involved in insulin initiation

In Australia the main health professional groups involved in insulin initiation are specialist physicians, specialist diabetes nurse educators (DNEs) and general practitioners (GPs). GPs are generalist physicians who work in the primary care setting. In other countries, practice nurses (nurses who work with and under the supervision of GPs) are involved in insulin initiation [10,19] and this practice nurse role is currently the focus of a cluster randomised controlled trial in Victoria, Australia [17]. In Australia practice nurses do not require any formal postgraduate qualification but have been required to meet continuing professional development standards since July 2010 [20]. Credentialed DNEs are also required to meet these standards, however they have also completed a Graduate Certificate course (1 year part time), 1800 hours experience in providing diabetes self management and education and have completed a mentoring program [21]. They may work in primary care, secondary care settings or both, but are considered specialists in their field. PwT2D require a referral from a GP to access specialist physician care. In this paper the term specialist physician refers to doctors who have completed specialty training either as an endocrinologist or general internal medicine physician.

Optimal management of T2D requires a multidisciplinary approach [22]. Regardless of the model used for supporting people to move on to insulin when needed, delivery of best practice care in the primary health setting requires collaborative practice and this is dependent on effective interprofessional relationships [23]. Coordination of care between health professionals in both general practice and secondary care settings may be important in obtaining the best outcomes for patients, particularly in providing the appropriate support for insulin initiation to become a routine activity within general practice. This paper describes the use of an organisational theory, relational coordination [24], to explore factors that may influence how health professionals could work together to commence PwT2D on insulin in the general practice setting.

### Relational coordination between health professionals involved in the initiation of insulin in PwT2D

In organisational theory, the study of relationships within and external to organisations and their work, coordination refers to the mechanisms which ensure the flow of information between people who play different roles in the division of labour. Extending the notion of coordination, relational coordination (RC) refers to a “mutually reinforcing process of interaction between communication and relationships, carried out for the purpose of task integration” [25]. Like inter-professional collaboration, RC has a focus on sharing, respect and communication between people in different roles of an organisation. In the health setting, both inter-professional collaboration and RC share the core values of high quality care enhanced by optimal communication across all members of the health care team, including the patient and their family [26]. RC theory, first developed by Gittell to explain the impact of role relationships on coordination and organisational outcomes in the airline industry, has now been applied in multiple health care settings, including primary care [27-32]. A survey tool has been developed to measure aspects of RC [24]. Understanding factors that impact on the degree of RC between particular professional roles is important because it goes beyond individual interactions. It allows understanding at a system and organisational level which may be useful for planning models of care within which health professionals may most effectively work and interact.

RC theory identifies key concepts that underpin effective interprofessional work: communication which is problem solving, timely, accurate and frequent which is dependent on relationships between professional roles, characterized by shared goals, shared knowledge and mutual respect [28]. This can be used as a framework for understanding the work of diabetes care in general practice, in particular the task of insulin initiation. Insulin initiation typically involves multiple health professionals working with a PwT2D to discuss the rationale for treatment, provision of a prescription and subsequent titration of the dose of insulin, patient education regarding insulin administration, and support of patients’ efforts at self management [22,33,34].

The aim of this study was to explore RC between specialist physicians, DNEs, GPs and practice nurses and the association between this and current practice for insulin initiation and reported appropriateness of this task being carried out in general practice. This study was intended to gain a range of opinions rather than to try to make generalisations about the health professional groups surveyed. We hypothesised that RC would be higher between specialists than between specialists and generalists, consistent with previous work which has shown lower levels of RC across organisational boundaries (e.g. secondary vs. primary care) [26]. We also expected higher RC between specialists and generalists to be associated with insulin initiation in general practice and with the belief that it is appropriate for insulin initiation to be carried out in general practice. Understanding the characteristics of inter-professional relationships is important as it is likely to shape the motivation and capacity of health professionals to work together to change clinical practice in this important area of diabetes management to provide timely care to PwT2D in the general practice setting.

## Method

### Participants

Surveys were distributed to specialist physicians, DNEs, GPs and practice nurses between August 2012 and March 2013. Multiple convenience methods of distribution were utilised. Paper based surveys (Additional files 1 and 2) were distributed in satchels at national conferences and at Victorian health professional meetings and education sessions as well as to professional networks of the authors. The survey was also available for completion electronically via an online survey (surveymonkey). The link to this electronic survey was distributed via national e-newsletters of health professional organisations and to professional networks of the authors.

### Measure

The survey consisted of three main components:

- Demographics: Information regarding occupation, number of years in practice and location of practice was collected. GPs and practice nurses were asked to identify whether they had previously been involved in the initiation and titration of insulin in the GP setting.
- Models of care for insulin initiation: Six models of care identified from a literature review were listed [35-39] (Table 1). Respondents were asked to indicate which model of care they currently worked within for PwT2D in General Practice who need to start insulin and then to rank the six models of care in order of perceived appropriateness, with 1 being most appropriate and 6 being least appropriate.

**Table 1:** **Descriptions of models of care**

**Model of care**	**Description**
General practice based care	Initiation and management of insulin by a GP +/- the assistance of a practice nurse
GP with a special interest in diabetes	GP that provides a clinical service beyond the scope of conventional general practice and can receive referrals from other GPs
Diabetes nurse educator (DNE)	Referral to a DNE to initiate and manage insulin in conjunction with a GP
Specialist shared care	Referral to a specialist (general physician or endocrinologist +/- DNE) for a one off consultation and provision of a management plan so that the GP can manage insulin
Specialist outreach	Referral to a specialist (general physician or endocrinologist +/- DNE) who conducts sessions within a general practice clinic
Specialist routine care	Referral to a specialist (general physician or endocrinologist +/- DNE) to take on primary responsibility of insulin initiation and ongoing management
- Relational coordination: This item consisted of seven survey questions which were adapted from a published measure of RC to specifically refer to the management of PwT2D in general practice who need to start insulin [40] (Table 2). Respondents were asked to rate their perceptions of the behaviour of other health professional groups, rather than reporting their own behaviour in order to reduce social desirability bias. These responses were measured on a five point scale and a composite score was calculated according to the method described by Gittell [24].

**Table 2:** **Relational coordination survey questions**

**Relational coordination domain**	**Survey question**
Frequent communication	How frequently do the care providers in each of these groups communicate with you about people with type 2 diabetes who are identified as requiring insulin in the general practice setting?
Timely communication	Do the care providers in each of these groups communicate with you in a timely way about people with type 2 diabetes who are identified as requiring insulin in the general practice setting?
Accurate communication	Do the care providers in each of these groups communicate with you accurately about people with type 2 diabetes who are identified as requiring insulin in the general practice setting?
Problem solving communication	When problems occur in people with type 2 diabetes who are identified as requiring insulin in the general practice setting, do the care providers in each of these groups blame others or work with you to solve the problem?
Shared goals	How much do the care providers in each of these groups share your goals for people with type 2 diabetes who are identified as requiring insulin in the general practice setting?
Shared knowledge	How much do the care providers in each of these groups know about the work you do with people with type 2 diabetes who are identified as requiring insulin in the general practice setting?
Mutual respect	How much do the care providers in each of these groups respect your work or role in people with type 2 diabetes who are identified as requiring insulin in the general practice setting?

Respondents were asked to respond to each question by rating GPs, practice nurses, DNE and physician on a 5 point Likert scale.

Paper surveys were returned at the conferences or via an enclosed reply paid envelope. Online surveys were completed via surveymonkey. The survey took less than 10 minutes to complete.

### Data analysis

Data was entered into an Excel spreadsheet, reviewed by a research assistant and then uploaded into Stata 12.1 statistical software (StataCorp, College Station, Texas, USA) for further cleaning and analysis. Complete case analysis was utilised for current models of care and ranking of appropriate models of care if no option was selected. Results were included in the analysis if at least one model of care was ranked. Descriptive quantitative analysis of the data was undertaken to describe survey respondents, current and preferred models of care and RC. Insulin initiation in general practice without specialist involvement was coded as being appropriate by respondents if GP or GP with a special interest in diabetes (GPwSI) care was ranked between 1 and 3. Fisher’s exact test was used to determine if this differed between the health professional groups. Cronbach alpha score of 0.89 was calculated, indicating that it was valid to aggregate the seven dimensions of RC into one index. Non parametric tests (Kruskal-Wallis equality-of-populations rank test with ties and Wilcoxon rank sum tests) were used to determine whether health professional type, model of care and belief that insulin initiation in general practice was appropriate impacted on RC scores. A symmetrical matrix of RC ties was developed in order to determine the strength of RC ties within and between each health professional group. These were then plotted on a radar graph.

#### Ethics

This study received ethical approval from the Human Research Ethics Committee (HREC) at the University of Melbourne (HREC ID: 1238199).

## Results

### Survey response

179 completed surveys were returned between August 2012 and March 2013. 144 were paper surveys and 35 were via surveymonkey (the electronic survey was accessed 52 times but not completed in 17 instances, which, whilst not formally assessed, may reflect a degree of responder fatigue).

### Demographics

The characteristics of the respondents are summarised in Table 3. The majority of health professionals worked primarily in major cities or inner regional areas. Practice nurses generally reported working in their role for a shorter period of time compared to the other health professional groups.

**Table 3:** **Demographics of survey respondents**

		**Physician**	**Diabetes nurse educator (DNE)**	**General practitioner (GP)**	**Practice nurse**	**Total**
Number returned (% of total respondents)		27 (15.1%)	62^1^ (34.6%)	46 (25.7%)	44 (24.6%)	**179**
Years in practice median (interquartile range)		12.5 (5.5-20)	10 (5-20)	22 (18-30)	5 (3-10)	
Setting worked within Number (% of health professional group)	Outpatients	18 (67%)	24 (38%)	1 (2.2%)	0 (0%)	
	CHC	1 (3.7%)	20 (32%)	7 (15%)	2 (4.7%)	
	Private	17 (63%)	15 (24%)	1 (2.2%)	3 (7.0%)	
	General Practice	0 (0%)	22 (35%)	38 (83%)	38 (88%)	
Primary location of work (RA level [57]^2^) Number (% of health professional group)	1	17 (63%)	29 (46%)	31 (67%)	30 (70%)	
	2	9 (33%)	21 (33%)	9 (20%)	9 (21%)	
	3	0	11 (18%)	5 (11%)	1 (2.3%)	
	4	0	0	1 (2.2%)	1 (2.3%)	
	5	0	0	0	1 (2.3%)	
Model of care most frequently worked within for initiation of insulin Number (% of health professional group)	General practice based care	1 (3.6%)	7 (11.3%)	22 (50%)	28 (65.1%)	
	GP with a special interest in diabetes	0	0	3 (6.8%)	0	
	Diabetes Nurse Educator	2 (7.1%)	42 (67.7%)	7 (15.9%)	10 (23.3%)	
	Specialist - Shared Care	8 (28.6%)	1 (1.6%)	8 (18.2%)	3 (7.0%)	
	Specialist- Outreach^3^	1 (3.6%)	0	0	2 (4.7%)	
	Specialist- Routine care	16 (57.1%)	12 (19.4%)	4 (9.1%)	0	

^1^Two respondents stated that they worked as both DNEs and practice nurses and were classed as DNEs for the purposes of analysis given their extended training.

^2^The Remoteness Area (RA) Classification system allows quantitative comparisons between city and rural Australia. The five RAs are: RA1- major cities, RA2-inner regional, RA3- outer regional, RA4- remote Australia and RA5- very remote.

^3^One physician indicated working most frequently in both outreach and routine care.

### Models of care for insulin initiation

The majority of specialist physicians indicated that they worked within a specialist routine model in which GPs referred PwT2D to them for primary responsibility of insulin initiation and ongoing management. 50% of GPs and 65% of practice nurses reported that the majority of initiation and management of insulin occurred in general practice, with or without the assistance of a practice nurse. The majority of DNEs reported initiating and managing insulin in conjunction with a GP (67.7%) (Table 3).

**Figure 1:**
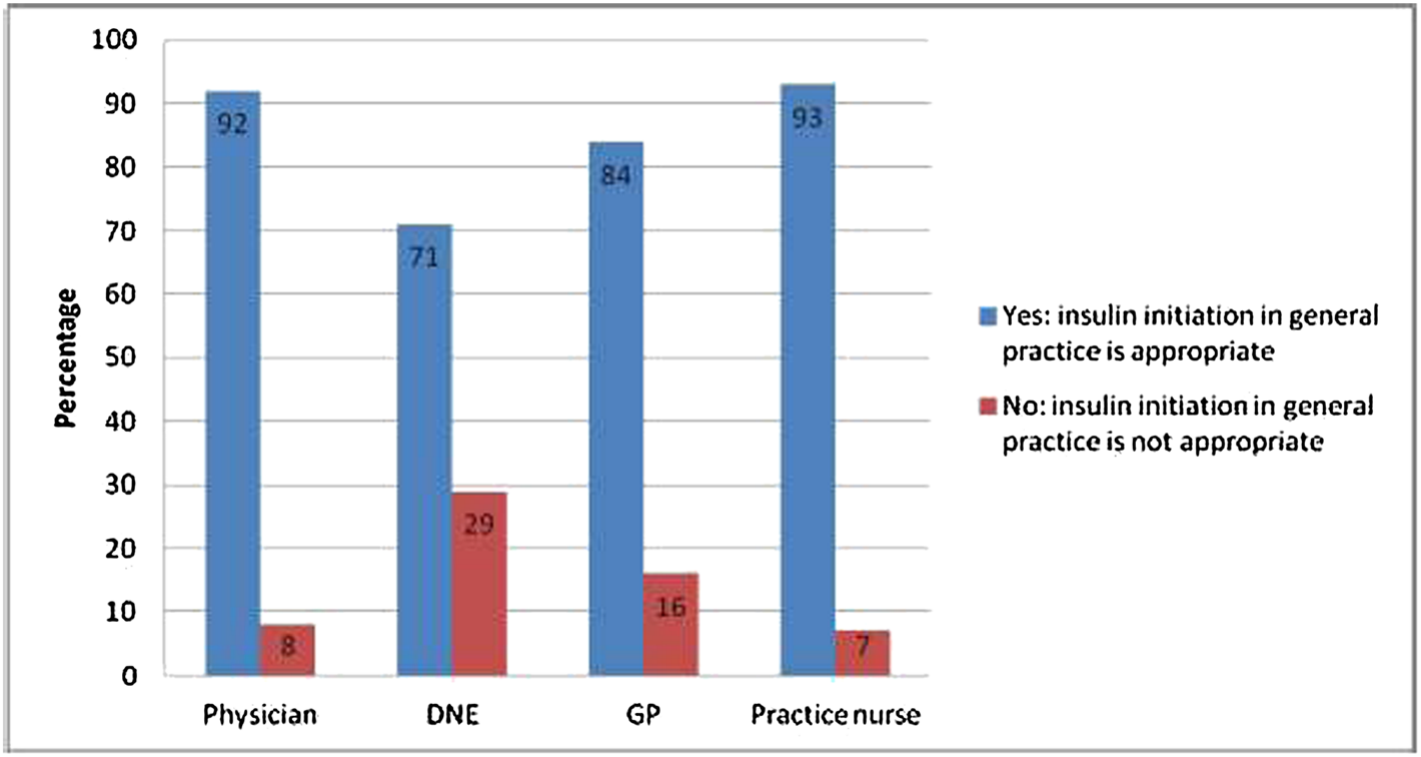
**Appropriateness of insulin initiation in general practice without specialist involvement.**

There was a significant difference in the extent to which each of the health professional groups viewed the different models of care as appropriate (p = 0.034). Over 90% of specialist physicians and practice nurses rated insulin initiation in general practice as appropriate as compared to 84% of GPs and 71% of DNEs (Figure 1).

### Relational coordination

**Figure 2:**
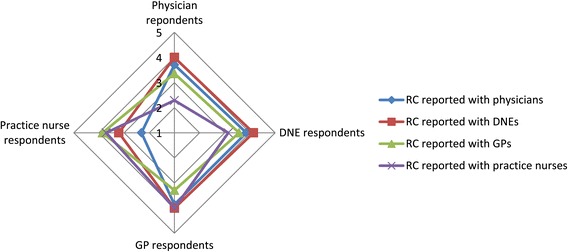
**Strength of relational coordination (RC) ties between professional groups.**

The highest median (IQR) RC was reported by GPs (3.79 (3.4, 4.1)) and the lowest by practice nurses (3.3 (3.0, 3.9). The median (IQR) RC reported overall in the study was 3.5 (3.2, 3.9). Specialist physicians reported stronger RC with other specialist practitioners (specialist physicians and DNEs), whilst practice nurses had stronger RC with those working as generalists in primary care (Practice nurses and GPs). With the exception of practice nurses, all health professional groups reported strongest RC with DNEs. DNEs lowest RC was with practice nurses whilst that for GPs was with their GP colleagues (Table 4 and Figure 2).

**Table 4:** **Relational coordination (RC) between health professional groups**

		**Physician**	**DNE**	**GP**	**Practice nurse**	**P value**
RC reported with Median (IQR)	Physician	3.71 (3.21, 4.07)	3.86 (3.29, 4.29)	3.86 (3.29, 4.14)	2.31 (1.67, 3.29)	0.0001
	DNE	4 (3.71, 4.14)	4.14 (3.71, 4.57)	4 (3.71, 4.29)	3.21 (2.43, 3.86)	0.0001
	GP	3.36 (3, 3.57)	3.57 (3.14, 4.14)	3.29 (2.57, 4)	3.86 (3.29, 4.57)	0.015
	Practice nurse	2.29 (1.83, 2.71)	3.14 (2.5, 3.5)	4 (3.17, 4.29)	3.71 (3.14, 4.57)	0.0001

### The relationship between RC and models of care for insulin initiation

The strength of RC reported with practice nurses was higher for health professionals that currently worked within a primary care based model of care for insulin initiation. In contrast, health professionals that worked within or referred to specialist focussed care reported stronger RC with DNE and specialist physicians. For DNE this related to the domains of frequent communication, timely communication, shared knowledge and mutual respect. For specialist physicians this applied to all RC domains with the exception of accurate communication. RC scores of all health professional groups in relation to GPs were not impacted by their reported current model of care.

### RC and belief that insulin initiation in general practice is appropriate

There was no association between the belief about the appropriateness of insulin initiation in general practice and the RC reported with nurses. However, there were some associations with RC domains reported with doctors. In particular, health professionals who felt that it was appropriate for GPs to initiate insulin also felt that GPs had a better understanding of their (i.e. the other professionals’) roles. These health professionals also reported significantly lower levels of frequent, timely, accurate and problem solving communication with specialist physicians.

## Discussion

### Insulin initiation in general practice is viewed as appropriate

This exploratory study demonstrated high levels of agreement from the four health professional groups surveyed that insulin initiation for PwT2D in general practice is appropriate, and is in agreement with research that indicates that it is safe and effective [41,42].

### Relational coordination is strongest within levels of care and is impacted by models of care

This study suggests that, as hypothesised, RC is strongest along specialist and primary care generalist lines respectively, particularly for specialist physicians and practice nurses. This finding is likely to reflect the fact that health professionals that work in the same organisation, physical location or level of care coordinate their work better than those who work in distant, separated organisations, and is consistent with previous RC studies [26].

Lower RC with specialists was reported by those working within general practice models of insulin initiation. Lower communication with specialist physicians was reported by those who saw these models as appropriate. Further work is indicated to explore whether these findings may be a cause or consequence of GPs taking a lead in initiating insulin. For example, low levels of RC with specialists may reflect a lack of relationships resulting in the need for the adoption of a general practice based model of care. Conversely, many of the primary healthcare professionals in this study already reported working in a general practice based model of care and may not feel a need to develop relationships and communication with specialists as they can manage insulin independently without them. If more insulin initiation is to occur in this setting it may be important to explore ways in which the current RC divide between specialist and generalist health care professionals can be improved. This may facilitate primary care professional’s access to specialist support to successfully incorporate insulin initiation as a routine general practice activity and to have timely access to resources for those patients who require a specialist level of care.

Specialist physicians reported lower RC with practice nurses and GPs relative to that reported with DNEs and their own professional group. This may reflect that the majority of specialist physicians in this study reported working primarily within a routine care model and as a result may have limited interaction with general practice outside of letters detailing referral and the outcome of consultation. This may not be an issue if GPs and practice nurses involved in insulin initiation have access to DNEs (with whom both GPs and specialist physicians report high RC) that can act as boundary spanners^a^ between the groups when required.

There is no benchmark data for RC in Australian general practice, however RC measured in this study is lower (median (IQR) 3.5 (3.2, 3.9)) than that reported in a recent study conducted in Danish general practice (4.1 ± 0.3) and in hospital studies (RC range 3.84 to 4.22). The lower RC observed in this study may be related to the focus on insulin initiation and the health professional sample surveyed working in different organisational settings (primary vs. hospital and specialist care). Potential barriers to RC across primary and specialist care include differing governance structures, different administrative practices, hierarchical organisational structures, and limited contact between health professionals due to time and geographical restrictions. These may impact on the ability to generate trust and knowledge of others’ roles [43]. Historical factors relating to professionalisation and gender, both between doctors and nurses and within these professional groups [44-52], present additional potential barriers which would benefit from further investigation in a qualitative study.

### Low relational coordination scores between practice nurses and DNEs may be reflective of a lack of interaction, and may limit practice nurses’ ability to expand their role in insulin initiation

Practice nursing is still developing in Australia and, compared to that for DNEs, there is no clear framework for education and career pathway and no consistent standards for the development of the practice nurse role [53]. This, combined with limited interaction between practice nurses and DNEs who are not working within a general practice model of care for insulin initiation, may have contributed to the lower RC reported between these professional groups. Work by Greaves investigating the needs of practice nurses in the United Kingdom in relation to insulin initiation found that practice nurses wanted DNE support and supervision, when required, as part of their training and ongoing support structure [54]. This study indicates that increased effort and resources may be required to facilitate communication and development of relationships between these two groups to enable this to occur.

### Strengths and limitations

This study adds to the literature for the use of RC in the primary care setting and is the first report of the use of the RC survey in an Australian health care study. RC may be an important consideration when developing models of care that rely on effective coordination between health professionals and this study demonstrates that work focussed on building relationships and communication between health professional groups may be important to develop effective models of care to facilitate increased insulin initiation in the general practice setting. There are three main limitations of this study which have implications for future work.

Firstly, respondents to the survey represented a convenience sample obtained through a targeted multi-methods approach, including attendance at conferences and education meetings which may indicate they were already involved in providing proactive patient care. In particular, a higher proportion of GPs and practice nurse respondents indicated that they initiate insulin within general practice without use of specialists than that previously reported in the literature [12]. This may have biased the responses as to the appropriateness of insulin initiation in general practice. Hence our findings may not be representative of the professions’ view at large. We collected limited demographic data and did not determine whether respondents were co-located with the health professionals listed in this survey. Missing or lower relational coordination scores reported with practice nurses may reflect limited interaction with practice nurses as previously described and may also reflect 40% of general practices not employing a practice nurse [55]. Many GPs and physicians work within group practices and so not providing a response to the RC with their respective groups may reflect low levels of collaboration and working in isolation.

This survey asked respondents to rate their perceptions of the behaviour of health professional groups rather than individuals. It may be difficult to rate groups within which there may be wide variation, particularly for those whose role isn’t well defined, can be variable, and is currently evolving and changing, particularly across organisations. In addition, such perceptions may not reflect the views of these health professionals accurately. This is an issue warranting further exploration, and may have been an issue particularly for the practice nurse group.

Finally, information related to gender and age may have been useful to explore given previous work that has indicated that gender may play an important role in relationships between doctors and nurses [48,52,56].

## Conclusion

This study has demonstrated that RC theory may be useful in exploring how health professionals work together to commence PwT2D on insulin in the general practice setting and for measuring the impact of interventions which aim to increase coordination between them. It suggests that building relationships and communication between specialist and generalist health professional groups, particularly between DNEs and practice nurses, may be important to facilitate the development of effective models of care to support insulin initiation in primary care. It also suggests a potential role for DNEs in acting as boundary spanners between primary and secondary care. Current practice, funding and models of care may impact on the ability to increase RC and this will form the basis of further research.

## Endnotes

^a^Boundary spanners “facilitate transactions and the flow of information between people or groups who either have no physical or cognitive access to one another, or alternatively, who have no basis on which to trust each other” [57].

## Additional files

Additional file 1:
**GP and practice nurse RC survey.**
Additional file 2:
**Specialist physician, DNE and GP RC survey.**

## Abbreviations

DNE: Diabetes nurse educator
GP: General Practitioner
PN: Practice nurse
PwT2D: People with type 2 diabetes
T2D: Type 2 diabetes
