# Supplementary material for: Relational coordination amongst health professionals involved in insulin initiation for people with type 2 diabetes in general practice: an exploratory survey
Source: BMC Health Serv Res. 2014 Nov 1;14:515. doi: 10.1186/s12913-014-0515-3 (PMC4224690; doi:10.1186/s12913-014-0515-3)
Supplement: Additional file 1: — GP and practice nurse RC survey. [file 12913_2014_515_MOESM1_ESM.pdf]

THE UNIVERSITY OF MELBOURNE

Insulin initiation in type 2 diabetes

A survey for GPs and Practice Nurses

What is this study about?

Currently the majority of care for people with type 2 diabetes occurs in the general practice setting. However, when insulin initiation is required it is generally not occurring in a timely manner and the majority of patients are referred to specialists (physicians and diabetes nurse educators (DNE)).

This survey is part of a project which will explore how health professionals (physicians, DNE, practice nurses and GPs) work together to commence people with type 2 diabetes on insulin and whether professional roles and models of care influence this. This survey is linked, and will occur in parallel, to the Stepping Up study (an NHMRC funded cluster randomised trial for insulin initiation in general practice in which the role of the practice nurse is enhanced) This study is important as it will determine the feasibility of the Stepping Up model and whether health professionals are willing to collaborate in order to increase the initiation of insulin within the general practice setting.

What will taking part in this study involve?

Taking part in the study will involve you completing this survey. **There are four pages in this survey. It will take less than ten minutes to complete. Please return it using the attached reply paid envelope or at the University of Melbourne Student-Led Clinic stall in the Exhibition Hall.** If you practice in Victoria and are interested in being interviewed about the way health professionals currently work together to commence people with type 2 diabetes on insulin in general practice please complete the contact details section later in the survey. If you would like to enter the prize draw for a $200 voucher, please fill in the relevant section of the survey.

Your participation in this study is voluntary. Consent to participate in the project will be implied by your return of the survey.

How will my privacy be protected?

If you do provide your name and contact details these will be detached and stored separately from the survey so that your responses will remain anonymous. Any research data collected will be kept confidential subject to legal requirements and maintained in accordance with The University of Melbourne guidelines for the conduct of research. All information will be kept secure in locked storage or on a secure password protected server at the Department of General Practice, The University of Melbourne. The de-identified research data will be stored for a maximum of five years after publication and destroyed thereafter.

Who can I talk to about this project?

This study will contribute towards a PhD thesis. The principal researcher is Dr Jo-Anne Manski-Nankervis who is being supervised by Dr John Furler and Dr Irene Blackberry at the Department of General Practice, The University of Melbourne.

If you have any questions about this study, please contact Dr Jo-Anne Manski-Nankervis, Department of General Practice, The University of Melbourne, ph (03) 9035 8019 or e-mail jomn@unimelb.edu.au.

This project has received clearance from the Human Research Ethics Committee at The University of Melbourne (HREC 1238199). If you have any concerns about the conduct of this project please contact the Executive Officer, Human Research Ethics, The University of Melbourne, ph (03) 8344 2073 or fax (03) 9347 6739.

The researchers gratefully acknowledge the RACGP Foundation for their support of this project.

Models of care

The aim of this part of the survey is to determine the current and preferred models of care for **people with type 2 diabetes who are otherwise well and are identified in the general practice setting as requiring insulin** in order to improve their glycaemic control.

Please complete the table below. Please only choose one option that you work within most frequently and then rank the models (from 1 to 6) that you think are appropriate for this patient group, where 1 = most appropriate and 6 = least appropriate.

Models of care for people with type 2 diabetes who are otherwise well and are identified in the general practice setting as requiring insulin	Tick the one model that you work within most frequently	Rank the most appropriate model for insulin initiation in this patient group from 1 to 6, where 1 = most appropriate, 6 = least appropriate.
**General practice based care**<br>Initiation and management of insulin by a GP +/- the assistance of the practice nurse	☐	☐
**GP with a special interest in diabetes**<br>GP that provides a clinical service beyond the scope of conventional general practice and can receive referrals from other GPs	☐	☐
**Diabetes Nurse Educator**<br>Referral to a diabetes nurse educator (DNE) to initiate and manage insulin in conjunction with the GP	☐	☐
**Specialist* – Shared Care**<br>Referral to a specialist (general physician or endocrinologist +/- DNE) for a one-off consultation and provision of a management plan so that the GP can manage insulin	☐	☐
**Specialist* Outreach**<br>Referral to a specialist (general physician or endocrinologist +/- DNE) who conducts sessions within a general practice clinic	☐	☐
**Specialist* – Routine Care**<br>GP Referral to a specialist (general physician or endocrinologist +/- DNE) to take on primary responsibility of insulin initiation and ongoing management	☐	☐

*The referral is made to an endocrinologist who may then involve a DNE in patient management

Collaboration

Circle a number in each box regarding each of the statements about collaboration.

1. Strongly disagree 2. Disagree 3. Neither agree nor disagree 4. Agree 5. Strongly agree OR N/A

Regarding the initiation of insulin for a person with type 2 diabetes who is otherwise well...	Physician / Endocrinologist	DNE	GP with special interest in diabetes	GP	Practice nurse
I am willing to collaborate with this group of health professionals	1 2 3 4 5 N/A	1 2 3 4 5 N/A	1 2 3 4 5 N/A	1 2 3 4 5 N/A	1 2 3 4 5 N/A
I trust this group of health professionals to provide high quality care	1 2 3 4 5 N/A	1 2 3 4 5 N/A	1 2 3 4 5 N/A	1 2 3 4 5 N/A	1 2 3 4 5 N/A
I respect this group of health professionals	1 2 3 4 5 N/A	1 2 3 4 5 N/A	1 2 3 4 5 N/A	1 2 3 4 5 N/A	1 2 3 4 5 N/A
I have good communication with this group of health professionals	1 2 3 4 5 N/A	1 2 3 4 5 N/A	1 2 3 4 5 N/A	1 2 3 4 5 N/A	1 2 3 4 5 N/A

Adapted from Relational Coordination survey www.rcrc.brandeis.edu

Please place a response for every professional group indicated, including how you interact with your own.

Frequent Communication

How **frequently** do the care providers in each of these groups communicate with you about **people with type 2 diabetes who are identified as requiring insulin in the general practice setting**?

When answering this question, be sure to consider all forms of communication, including in-person meetings, phone calls, e-mails, etc.

	Never	Rarely	Sometimes	Often	Always
General Practitioner	☐	☐	☐	☐	☐
Practice Nurse	☐	☐	☐	☐	☐
Diabetes nurse educator	☐	☐	☐	☐	☐
Physician	☐	☐	☐	☐	☐

Timely Communication

Do the care providers in each of these groups communicate with you in a **timely** way about **people with type 2 diabetes who are identified as requiring insulin in the general practice setting**?

When answering this question, be sure to consider all forms of communication, including in-person meetings, phone calls, e-mails, etc.

	Never	Rarely	Sometimes	Often	Always
General Practitioner	☐	☐	☐	☐	☐
Practice Nurse	☐	☐	☐	☐	☐
Diabetes nurse educator	☐	☐	☐	☐	☐
Physician	☐	☐	☐	☐	☐

Accurate Communication

Do the care providers in each of these groups communicate with you **accurately** about **people with type 2 diabetes who are identified as requiring insulin in the general practice setting**?

When answering this question, be sure to consider all forms of communication, including in-person meetings, phone calls, e-mails, etc.

	Never	Rarely	Sometimes	Often	Always
General Practitioner	☐	☐	☐	☐	☐
Practice Nurse	☐	☐	☐	☐	☐
Diabetes nurse educator	☐	☐	☐	☐	☐
Physician	☐	☐	☐	☐	☐

Problem Solving Communication

When problems occur in **people with type 2 diabetes who are identified as requiring insulin in the general practice setting**, do the care providers in each of these groups blame others or work with you to **solve the problem**?

	Always blame	Mostly blame	Neither blame nor solve	Mostly solve	Always solve
General Practitioner	☐	☐	☐	☐	☐
Practice Nurse	☐	☐	☐	☐	☐
Diabetes nurse educator	☐	☐	☐	☐	☐
Physician	☐	☐	☐	☐	☐

Shared Goals

How much do the care providers in each of these groups **share your goals** for **people with type 2 diabetes who are identified as requiring insulin in the general practice setting**?

	Not at all	A little	Somewhat	A lot	Completely
General Practitioner	☐	☐	☐	☐	☐
Practice Nurse	☐	☐	☐	☐	☐
Diabetes nurse educator	☐	☐	☐	☐	☐
Physician	☐	☐	☐	☐	☐

Shared Knowledge

How much do the care providers in each of these groups **know** about the work you do with **people with type 2 diabetes who are identified as requiring insulin in the general practice setting**?

	Nothing	Little	Some	A lot	Completely
General Practitioner	☐	☐	☐	☐	☐
Practice Nurse	☐	☐	☐	☐	☐
Diabetes nurse educator	☐	☐	☐	☐	☐
Physician	☐	☐	☐	☐	☐

Mutual Respect

How much do the care providers in each of these groups **respect** your work or role in **people with type 2 diabetes who are identified as requiring insulin in the general practice setting**?

	Not at all	A little	Somewhat	A lot	Completely
General Practitioner	☐	☐	☐	☐	☐
Practice Nurse	☐	☐	☐	☐	☐
Diabetes nurse educator	☐	☐	☐	☐	☐
Physician	☐	☐	☐	☐	☐

Some questions about you…

Are you a:

☐ GP

☐ Practice Nurse

How many years have you been in practice? ________ years

What is the postcode of your primary location of practice? ☐ ☐ ☐ ☐

Please tick which setting/s you currently work within:

☐ Community health centre

☐ Private billing general practice

☐ Bulk billing general practice

☐ Mixed billing general practice

☐ Other (PLEASE STATE): ______________________________

Have you been involved in the initiation and titration of insulin within the general practice setting?

☐ No

☐ Yes — If yes, what was your role?

☐ Education

☐ Titration of insulin

☐ Other (PLEASE SPECIFY): ____________________

Thank you for completing this survey. Please return the survey in the attached reply paid envelope or to the University of Melbourne Student-Led Clinic stall in the Exhibition Hall.

ID No. ☐ ☐ ☐

Prize Draw and Expression of Interest for Interviews

☐ I would like to enter the draw for a $200 Coles Myer voucher

Please provide your details →

☐ I consent to being contacted to be interviewed for this study

Please provide your details →

Name:

Phone no:

E-mail:

Best time to contact

Day(s):

Time(s):

If you do provide your name and contact details these will be detached on receipt of the survey and stored separately so that your responses will remain anonymous
